# Pure Juice Supplementation: Its Effect on Muscle Recovery and Sports Performance

**DOI:** 10.21315/mjms2023.30.1.4

**Published:** 2023-02-28

**Authors:** Siti Maizura Mohd Daud, Nursyuhada Mohd Sukri, Mohamad Hanapi Johari, Justin Gnanou, Faizal Abdul Manaf

**Affiliations:** 1 Defence Fitness Academy, National Defence University of Malaysia, Kuala Lumpur, Malaysia; 2 Faculty of Medicine and Defence Health, National Defence University of Malaysia, Kuala Lumpur, Malaysia; 3 School of Medicine, International Medical University, Kuala Lumpur, Malaysia

**Keywords:** sports nutrition, antioxidant, fruit juice, delayed-onset muscle soreness, exercise performance

## Abstract

Strenuous exercise causes increased production of reactive oxygen species (ROS), creating an imbalance between ROS and antioxidants. The reduced antioxidant defence leads to defective elimination of ROS and consequently, delayed-onset muscle soreness (DOMS). DOMS due to exhaustive or prolonged exercise typically peaks between 24 h and 72 h after exercise results in soreness, inflammation, pain and decreased muscle function. As a result, muscle strength will be reduced progressively and this situation might be detrimental to one’s athletic performance, especially amidst competition season. Therefore, supplementation to improve muscle recovery and sports performance has become a common practice among athletes. However, it is suggested to consume natural-based fruit-derived antioxidants as a more effective and safe nutritional strategy. Fruits containing a high amount of polyphenol protect muscle cells from excessive and harmful ROS due to their anti-inflammatory and antioxidant characteristics. To date, there are several expended studies on the consumption of supplements from various antioxidant-rich fruits to provide evidence on their effectiveness, giving better solutions and wider choices of supplementation to the athletes. Therefore, this review aims to provide a comprehensive overview of nutritional standpoint from previous literature on the effect of fruit juices supplementation on muscle recovery and sports performance.

## Introduction

Exhaustive and strenuous exercise has been shown to cause skeletal muscle damage and has been implicated as one of the factors leading to delayed-onset muscle soreness (DOMS) (1, 2). Strenuous exercise might also increase reactive oxygen species (ROS) production, leading to an imbalance between antioxidants and ROS (3). The reduced antioxidant defence might affect the elimination of ROS, leading to DOMS (4). DOMS following exercise depends on the type and the severity of exercise and usually peaks between 24 h and 72 h after exercise (5). This period is crucial for athletes during training and competition when there is a limited duration for recovery (2).

Exercise-induced skeletal muscle damage (EIMD) is a common complication of strenuous physical activity. Many studies have found that instantly after strenuous physical activity, biomarkers of skeletal muscle damage such as lactate dehydrogenase (LDH), myoglobin and creatinine kinase (CK) levels are increased significantly, often more than 5-fold–100-fold (6–8). A study by Park et al. (7) observed athletes for a longer duration after a race found that these skeletal muscle damage biomarkers will return to normal after 7 days–10 days with proper post-race recovery. However, these damages may affect their performance and limit their potential during events since they only have a limited time allocation for recovery.

Friden et al. (9) explained the complex mechanism of muscle damage. It was described as a mechanical imbalance between adjacent sarcomere, the breakdown of the sarcotubular system, myofibrillar disruption, cytoskeletal damage and defect on the extracellular myofiber matrix. According to Owens et al. (1), the postulated mechanism can be summarised into two phases. First, the mechanical work that causes the primary damage and second, the inflammatory response processes propagating tissue damage and causing secondary damage. The primary damage due to prolonged mechanical load caused coupling failure of the sarcomere and sarcolemma from eccentric lengthening and excitation-contraction (E–C) (1, 10, 11). The primary phase triggered the cascade of the secondary phase, where there is an influx of Ca^2+^ into the cytoplasm to maintain homeostasis causes further damage (12–14). In this light, the secondary cascade increased the production of ROS and oxidative stress, which causes DOMS (12).

Exercise-induced muscle damages, which cause inflammatory responses, are common among athletes during prolonged training or competition. Antioxidant and anti-inflammatory nutritional supplements are common strategies used to reduce these damages (15). Theoretically, consuming antioxidants will help inhibit ROS formation. This is because antioxidants will bind to metal ions, reduce hydrogen peroxide formation, quench the superoxide and singlet oxygen, and break the chain reaction (3, 16–18). This process will improve muscle recovery and performance as it helps reduce inflammatory responses, which cause muscle damages.

Nowadays, various kinds of supplementation for muscle injury, recovery and performance are available in the market. However, the US Food and Drug Administration (FDA) has warned that these supplements might contain additive and dangerous substances which could cause unwanted side effects. Previous studies have shown that about 38%–90% of athletes or the active population consume supplements despite a lack of scientific evidence to support their effectiveness in boosting performance and recovery (19–21). Therefore, it is crucial to recognise effective nutritional strategies to encourage faster recovery between intense training periods at pre-season, during 1 day training sessions and between competitions during competition or tournament seasons.

In general, antioxidants can be obtained from fruits and vegetables containing polyphenols. Close et al. (22) stated that inflammation and oxidative damage within the muscle causes fatigue and decreased performance due to excess ROS attenuated by the consumption of fruit-derived polyphenols with antioxidant and anti-inflammatory properties. Furthermore, the daily consumption of >1000 mg polyphenols for at least 3 days prior to and following exercise or approximately 300 mg polyphenol an hour prior to exercise may ameliorate muscle perfusion, enhancing muscle recovery and performance (17). Therefore, this paper aims to summarise the latest empirical evidence on the effect of an antioxidant-rich supplement derived from fruits juices to offer more natural-based nutritional solutions for skeletal muscle recovery and improved sports performance among athletes.

### Fruit Juices Supplementation

The Codex Alimentarius Commission (CAC) (23), established by the United Nations, Food and Agriculture Organization (FAO), defined fruit juice as ‘unfermented yet fermentable liquid obtained from the edible portion of the sound, sufficiently mature and fresh fruit or of fruit preserved in sound condition by suitable means like post-harvest surface treatments applied in compliance with the relevant provisions of the Codex Alimentarius Commission’.

According to the CAC, there are four forms of fruit juice in the market: i) simple juices, produced by just pressing and consisting of 100% fruit; ii) up to 50% condensed juices, which a large quantity of water is removed; iii) dehydrated juices which are condensed to powder and iv) nectarine juices, which in addition to juice, often contain 30%–50% pulp and added with sugar dissolved in water. It was also stated that the processes used to make a juice must maintain the physical, chemical, organoleptic and nutritional properties of the juices from the fruit it originates. A single juice is directly derived from one fruit or the same type of fruit by reconstituting the condensed juice. Meanwhile, a mixed juice is produced by combining two juices or more or a combination of juices and purées from various types of fruit (CODEX STAN 247-2005). In general, fruit juice can be described as an extract or an extractable liquid content of cells or tissues formed by mechanically pressing or squeezing out the natural fluid contained in ripe fruits without using any solvent or heat (24).

The global market for juices is rising and is possibly influenced by the health-conscious consumer and the desire for healthier food items. In 2015, the Transparency Market Research Report reported that juice producers are now more customer-centred and concentrate on launching various juices, mixed juices and flavours with creative packaging and extensive diet and health statements. Due to various factors, their consumption has increased globally in recent years. Another reason is their nutritional quality; despite being high in antioxidants, polyphenols, vitamins, fibre and moisture, they are low in fat, proteins and minerals. Hence, juice consumption will possibly help to prevent dietary deficiencies (25).

Besides containing essential nutrients, fruit juices also contain 80%–89% water (26). Water is a source of mineral salts and plays a significant function as a diluent of orally absorbed substances, including drugs, when used as a drink that facilitates digestive processes. Moreover, healthy individuals have a constant water balance because the water loss through the skin, respiratory tract, urine and stool will be replaced through water absorption from beverages, consumption of foods and tissue metabolism (27). Besides macronutrients and micronutrients, fruit juices are also rich in nutraceutical compounds which could promote immunity and bring other diverse health benefits (24).

Staying hydrated by drinking while exercising is an effective nutritional strategy that greatly affects performance. Furthermore, athletes also consume ‘liquid fuel’ before, during and immediately after exercise to maximise exercise gains and improve performance (28). Liquid fuel contains high carbohydrates but has low fat, protein and fibre content, making it a good choice for sports supplementation. It can be concluded that sports supplementation in a beverage, such as fruit juices, is the best option to meet an athlete’s nutrients demand during training or competition.

## Nutritional strategy for muscle recovery and performance using fruit juice

Various studies have been conducted using antioxidant-rich natural fruits juices to investigate the potential relationship between ROS, DOMS and sports performance. Theoretically, antioxidants help boost antioxidant defence to reduce oxidative stress by preventing or lowering the release of ROS after strenuous exercise. This helps reduce DOMS and promote improved performance during competition or strenuous exercises (29). In this review, articles on quantitative experimental studies on the use of fruit juices as a supplement for muscle recovery and sports performance among athletes or the physically active population published between 2010 and 2020 were chosen. Studies focusing on the effects of natural fruit juices supplements on muscle recovery and sports performance are summarised in Table 1.

### Beetroot Juice

Dietary nitrate (NO^3−^) supplementation has become popular among athletes due to its proven function to increase mitochondrial efficiency, glucose homeostasis, respiration, muscle contractility and vascular function (30, 31). Beetroot has a concentration of nitrates and other phytochemicals, such as betalains, making it rich in bioactivity compounds (32, 33). In recent years, beetroot juice has become one of the most recommended natural supplements for sports performance among athletes.

A recent study by Daab et al. (34) involving 13 semi-professional soccer male players found that daily supplementation of natural beetroot juice (2 × 150 mL/day) on the day of exercise and 3 days post-exercise enhanced muscle recovery after a simulated soccer match. Although there is no difference between the beetroot juice supplemented group and the placebo group regarding blood markers of muscle damage, the study reported a lower DOMS perception score among athletes who consumed the beetroot juice supplement. Similarly, Clifford et al. (35) demonstrated attenuated muscle pain and reduced dynamic muscle function after consuming beetroot juice supplement (250 mL or 125 mL) for 3 days following a muscle-damaging repeated sprint test (RST). However, there is no evidence of improvement in sprint performance and isometric strength recovery. Nevertheless, the data indicate that beetroot juice could be used as a post-exercise recovery solution to reduce injuries between bouts of repeated sprint exercises in certain aspects of dynamic muscle function among team-sports athletes. However, since sprint performance was unaffected, there is still uncertainty on whether these results are applicable in an actual team-sport competition. Future studies are required to understand the underlying cellular mechanisms. Several studies found that the benefits of beetroot juice are not related to other biochemical markers of muscle damage and changes in systemic oxidative stress.

On the other hand, Clifford et al. (36) reported that consuming beetroot juice (3 × 250 mL/day) for 3 days after a marathon does not reduce muscle pain, increase muscle function recovery, or attenuate inflammation and muscle damage biochemical markers. In this regard, the result does not support the use of beetroot juice as a treatment for muscle recovery among elite runners. Another study by Clifford et al. (37) discovered that muscle function recovery or attenuation of creatine kinase (CK) efflux or high-sensitivity C-reactive protein (hs-CRP) after exercise were not impaired by acute beetroot juice and sodium nitrate supplementation (250 mL or 125 mL) at immediately after exercise, 24 h and 48 h post-exercise. In the meantime, the study found that beetroot juice is more useful for attenuating the pressure-pain threshold (PPT) than sodium nitrate and suggests that it may be useful for those frequently engaging in sports or strenuous physical activity. The findings also revealed that the phenolic and betalains are the two compounds most likely to show analgesic effects in beetroot juice after exercise. Based on these findings, further exploration of the athletes’ pain relief ability is required when doing sports or exercising and also in clinical settings.

Clifford et al. (38) suggested that supplementation with antioxidant-rich beetroot juice (250 mL or 125 mL) at immediately after exercise, 24 h and 48 h post-exercise significantly improves several features of post-workout recovery including muscle soreness. Therefore, athletes seeking nutritional strategies to increase their antioxidant intake should consume beetroot juice or other antioxidant-rich functional foods rather than high doses of vitamins C and E supplements that may interfere with muscle adaptations when exercising.

### Grape Juice

Grapes are known to be high in antioxidants and bioactive properties. Studies have investigated the potential of grapes in causing an ergogenic effect in athletes compared to other fruit juices (39). Compared to other supplements, grape juice is cheaper, contain higher antioxidants and carbohydrates, and could not be contaminated by substances prohibited by the World Anti-Doping Agency (WADA) (40).

Studies conducted on grape juice supplementation among athletes have shown a positive ergogenic effect on performance and reducing blood markers of muscle damage. This is demonstrated by the latest study on the effect of grape juice intake (400 mL/day) for 2 weeks among 12 male volleyball athletes on oxidative stress and inflammation (40). During the experimental trial, grape juice supplementation consumed pre-exercise minimised DNA damage and protein oxidation by sporadic physical activity without epigenetics. The other two studies conducted by the same researcher also supported this finding. Toscano et al. (39, 41) found that grape juice supplementation (10 mL/kg/day) in both studies at 1 day and 28 days pre-exercise among recreational runners has an ergogenic effect by improving time-to-exhaustion, as supported by the significant reduction in inflammatory markers and higher antioxidant activity.

Studies found that grape juice has natural antioxidant and anti-inflammatory effects. Thus, it could be an economical option for athletes who want to avoid consuming over-processed food supplements. Grape juice may be considered a complete pre-workout food for athletes as it has a high caloric value due to its composition of carbohydrates (39). The recommendation of whole grape juice as a potentially ergogenic food for recreational athletes is a realistic outcome of these studies. In this light, grape juice can be considered an excellent choice for everyday consumption or even ingestion by athletes’ pre-competition.

### Pomegranate Juice

Pomegranate is considered a powerful and functional food rich in polyphenols, primarily containing ellagitannins and their related metabolites, which can defend against most forms of free radical oxidants (42). Current evidence indicates that supplementation with pomegranate juice can promote anti-inflammatory and antioxidant activity during and after exercise, increase strength and endurance performance and recovery after exercise, and improve cardiovascular responses while exercising (43).

A study conducted by Ammar et al. (44) on weightlifting training showed the positive impact of pomegranate juice intake on oxidative stress with consumption of three times 250 mL dose per day at 2 days pre-exercise and 500 mL at 1 h pre-exercise. This study has proven that pomegranate juice supplementation 48 h prior to the training could enhance antioxidant responses and attenuate oxidative stress. The findings of this study are related to a study by Ammar et al. (45) which reported that consumption of pomegranate juice with the same dosage and timing before weightlifting session prolonged the responses of muscle damage and inflammation, reduced the acuteness of the pain, stimulates recovery and increases weightlifting performance. These findings suggested that elite weightlifters could consume natural pomegranate juice to assist in muscle recovery during rigorous training and competition. They might benefit from the oxidative stress response that could provide faster recovery between training sessions.

Pomegranate juice supplementation was also studied by Trombold et al. (16) with significant and positive results. It was found that pomegranate consumption of 250 mL dosage at 12 h interval for 7 days pre-exercise, on the day of exercise and 7 days post-exercise has a protective effect against muscle soreness and fatigue after elbow flexor muscle exercise repetitions among 17 trained male athletes. However, it does not affect the knee extensor muscles. These findings suggest pomegranate juice supplementation has a moderate, acute ergogenic effect after eccentric exercise in the elbow flexor muscles of individuals with resistance training. The same research group conducted a study involving 16 recreationally active males and found that while the intake of 500 mL pomegranate extract at 12 h interval for 4 days pre-exercise, on the day of exercise and 4 days post-exercise decreases muscle soreness, it does not affect CK, Mb, interleukin-6 (IL-6) and CRP (46). This study focused on the ellagitannins content in pomegranate extract, which substantially enhances recovery of isometric strength 2 days–3 days after a strenuous eccentric exercise.

Two studies by Ammar et al. (44, 45) involved physically active individuals who did not perform any weightlifting-type exercises. Both studies found that pomegranate juice supplementation (750 mL/day at 2 days pre-exercise and 500 mL at 1 h pre-exercise) has a positive effect on both trained and untrained muscles. Therefore, besides trained athletes, pomegranate juice supplementation could be consumed by people in physically active professions, such as policemen, soldiers, firefighters, emergency workers and construction workers. This supplementation will be useful in increasing performance even when they are not trained in eccentric muscle contraction in the past 6 weeks (46).

Another study conducted among 45 recreationally active males by Machin et al. (47) using different doses of pomegranate juice (1 × 30 mL/days or 2 × 30 mL/days) at 3 days pre-exercise, on the day of exercise and 4 days post-exercise has shown that no dose-response effect presented. However, it is clear in this study that pomegranate juice supplementation promotes strength recovery after eccentric exercise in leg and arm muscles. It can be concluded the amount of daily intake of the pomegranate juice (once or twice daily) does not cause any differences in strength recovery after eccentric exercises. Nevertheless, the positive effect of pomegranate juice supplementation is more apparent when the juice contains > 0.7 g total polyphenol per 0.5 L, when larger muscle mass is involved and when it is ingested 60 min before exercising (43). Therefore, further studies are needed to validate these findings using more various and bigger sample sizes and determine the dosage, timing and frequency of pomegranate juice supplementation that may provide physiological and performance adaptations to refine the recommendations.

### Tart Cherry or Montmorency Cherry Juice

The nutraceutical market is showing rapid growth and it is necessary to identify for whom these items may be useful. Natural anti-inflammatory substances are used for decades to resolve the inflammation process with fewer complications. One example of these substances is tart cherry juice. Tart cherry, also known as Montmorency cherry, is one of the foods that recently garnered interest due to its potential in treating muscle damage. It has been promoted by lay publications as a high antioxidant and anti-inflammatory functional food beneficial for arthritis, muscle pain and fibromyalgia patients (48). In addition, tart cherries tend to have similar effects in reducing the inflammatory reaction in both acute and chronic pain syndromes experienced by athletes and non-athletes with chronic inflammatory disorders (49). Several studies have investigated the effect of tart cherry juice supplementation containing antioxidants on inflammation, muscle recovery and performance with mixed results.

One of the studies showed a positive effect on muscle recovery when tart cherry juice (2 × 237 mL/day) was taken for 5 days prior to, on the day of and 2 days after running a marathon by reducing lipid peroxidation and inflammation, improved total antioxidant capacity, and reduced CRP, IL-6 and uric acid (UA) levels (15). This finding is also supported by Kuehl et al. (48) which investigated the effect of consuming tart cherry juice (2 × 355 mL/day) 7 days prior to and during a strenuous running event among 54 healthy runners. The study showed that the tart cherry juice supplementation regime could minimise post-run muscle pain symptoms among the subjects in a robust endurance running event. In a subsequent study, the effect of tart cherry juice blend on muscle recovery among 16 male trained cyclists was investigated in the same laboratory settings (50, 51). The study findings indicated that consumption of tart cherry juice (2 × 30 mL/day) at 4 days pre-exercise and 3 days on the day of exercise might be an efficient functional food for boosting recovery and reducing cascades of exercise-induced inflammation that causes cellular disruption after strenuous cycling exercises. Bowtell et al. (52) also supported these findings. The study found that consuming tart cherry juice supplementation (2 × 30 mL/day) for 7 days prior, a day during and 2 days after a knee extensor resistance exercise improved the recovery of isometric muscle strength. This result was probably due to the antioxidative and anti-inflammatory properties of the juice in reducing oxidative stress.

Contrary to the studies mentioned above, a study conducted by McCormick et al. (53) on tart cherry juice intake (90 mL/day) 6 days pre-exercise among nine male Water Polo athletes found no significant effect on their muscle recovery and water polo performance. This might indicate that tart cherry juice supplementation has no benefit in improving muscle recovery and performance and is unnecessary for non-weight bearing intermittent-demand sports like Water Polo. Nonetheless, future studies are suggested to investigate the effect of tart cherry juice consumption in other running-based weight-bearing sports as an effective method to promote recovery and performance.

Evidence of the marked reduction in inflammation by tart cherry juice supplementation might provide a viable alternative solution to medicinal and therapeutic intervention and useful in aiding recovery following multiple training sessions among athletes with a relatively short recovery time allocation. There is a need for more studies to explore serum biomarkers and the possible explanation for reducing pain and inflammation associated with the consumption of tart cherry juice, and further studies should focus on the interaction of tart cherry, oxidative stress, inflammation and human performance by measuring different range of performance (50). This may help to extend the application to other sports with higher metabolic stress, such as team invasion sports.

### Other Fruit Juices

An interesting study conducted by Boussetta et al. (54) combined two variables: orange juice supplementation and air quality to investigate their effects on performance, cardiovascular parameters, muscle damage and oxidative stress among 11 male soccer players. This study followed a randomised, single-blind, crossover design. The subjects underwent two separate trials conducted in the evening in areas with polluted and non-polluted air 2.5 h after consuming 500 mL of fresh red-orange juice. During the trial in the polluted area, the football players’ CK and malondialdehyde (MDA) levels decreased significantly after consuming orange juice supplementation. It can be concluded that consuming red-orange juice could be an effective strategy to reduce the risk of pollution, especially in relation to muscle damage and oxidative stress markers. Therefore, all sports practitioners need to be sensitive to air quality while doing exercises, especially during the evening.

Other than that, supplementation of berries rich in polyphenol and antioxidants gives health benefits is strongly supported by other studies. For example, a study on the consumption of acai berry juice blend (100 mL/day) in 6 weeks involving seven junior hurdlers observed a marked improvement in lipid profile, total antioxidant capacity and a small reduction of DOMS. However, no effect was observed on sprint performance (55). Another study on New Zealand blueberry smoothie supplementation (200 g blueberry mixed with 50 g banana and 200 mL apple juice) among 10 recreationally active females shows a positive effect on the recovery of muscle peak isometric strength. The finding strongly supports the idea that its supplementation induces cellular adaptive events that accelerate muscle recovery of muscle isometric strength (56). These findings may benefit sports practitioners considering dietary strategies that target health and performance adaptation by using antioxidant-rich fruits such as berries.

A study on the use of watermelon juice as a muscle recovery supplementation (500 mL at 1 h pre-exercise) also found a beneficial impact on muscle soreness (57). The in vitro study also stated that non-pasteurised watermelon juice has greater bioavailability of natural non-essential amino acid, L-citrulline, which acts as the functional compound in controlling muscle fatigue. Hence, watermelon juice is a potential fruit juices supplement for muscle recovery. Another study by Tsitsimpikou et al. (58) focused on administering tomato juice supplements on 15 anaerobically trained athletes. The study revealed a favourable effect on muscle recovery by lowering the levels of lactate dehydrogenase (LDH), creatine phosphokinase (CPK) and CRP. Furthermore, the study suggested that tomato juice intake (~100 g/day) for 2 months during and post-exercise could significantly alter a subject’s muscle damage and inflammation adaptation due to anaerobic training regards. The benefits of antioxidants rich different fruits were also proved through a study on the anthocyanin-rich juice consumed (2 × 240 mL/day at 12 h interval) for 4 days before, at the day and 4 days after downhill running for 30 min at 70% VO_2max_. The study involved 30 healthy young males (59). This research demonstrates improved recovery of running economy and muscle function and attenuated muscle soreness, and significant reduction of CK levels.

Although fruits contain a high value of antioxidants and anti-inflammatory compounds, they might also cause detrimental effects on muscles recovery. Unexpected preliminary results from a study by Lynn et al. (60) proved that consumption of Billberry juice (2 × 200 mL/day) 5 days prior, on the day of and 2 days after a half-marathon among recreationally trained runners negatively affects muscle damage by increasing the exercise-induced exercise DOMS and CRP in a small to moderate value. However, the researcher has suggested a more wide-scale study to confirm the temporary adverse changes observed during the study. Despite the sound evidence of antioxidants’ effectiveness on muscle recovery, caution is still required when preparing for a long-term antioxidant supplementation intervention as it may blunt training adaptations (59). Moreover, in-depth studies are needed to understand the underlying mechanism of antioxidant supplementation towards muscle recovery.

## Conclusion

In conclusion, there is increasing evidence that various polyphenol-rich or antioxidant-rich supplements could effectively lower muscle damage and improve sports performance with the mechanisms most likely to be associated with antioxidant and vascular impact. However, based on the literature review, there is still limited evidence on their ergogenic effects. The rationale for these ambiguous findings could be the variations in each individual’s psychological and physiological responses toward each supplementation. For instance, there could be differences in the participants’ body systems regarding storage, absorption and transportation of active ingredients in the supplement. Other than that, differences in individual physical characteristics, such as body composition, fitness level, training levels, age and sex, could affect various responses towards the supplement being consumed. The type, timing and dosage of supplements given also varied.

The types of exercises conducted in the experiments are also different, which may give distinctive results. This is because DOMS is also affected by the types of muscle involved and the intensity and duration of exercise. However, despite these contrasting findings, it appears that antioxidants supplementation does have a beneficial effect on recovery and performance. In this context, fruit juices could be the best natural-based food supplements as they are rich in antioxidants, replacing other supplements products in aiding muscle recovery and improving the sports performance of trained athletes. Future research on fruit juice supplementation in terms of optimal dosage, timing of consumption, and frequency of ingestion would help better understand their effectiveness on recovery measures, including athlete performance and indices of muscle damage (17). However, caution is warranted when preparing long-term antioxidant supplementation, as training adaptations could be hindered.

## Figures and Tables

**Table 1:** Studies on the effect of fruit juices supplementation on muscle recovery and sports performance

Study	Supplementation protocol	Exercise protocol	Study population and design	Findings			

				Muscle recovery		Exercise/sports performance	
	
				Marker	Effect	Test	Effect
Daab et al. (34)	Beetroot juice^a^ 2 × 150 mL/d 3 d pre-ex 1 d the day of ex. 3 d post-ex	3 × CMJ, 3 × SJ, MVC at 90° knee flexion, 20 m SP and LIST	13 semi-professional soccer male players *Randomised, double-blind, crossover*	DOMS score	↓ 0 h and 24 h after ex	CMJ	↑ (24 h, 48 h and 72 h) ↑ (0 h, 24 h and 48 h) ↑ (48 h)
				CK	↔		
				LDH	↔	MVC	
				CRP	↔		
				CRO	↔.	SP	
Clifford et al. (36)	Beetroot juice^a^ 3 × 250 mL/d 3 d post-ex	Marathon running + MIVC + CMJ at 90° knee flexion + vertical jump max force	34 marathon athletes *Double blind, independent groups*	CK	↔	CMJ	↔
				IL-6	↔	MIVC	↔
				IL-8	↔		
				TNF- α	↔		
Clifford et al. (37)	Beetroot juice^b^ 2 groups: 250 mL OR 125 mL 3 × immediately post-ex 2 × 24 h post-ex 2 × 48 h post ex	100-drop jumps (0.6 m) + MIVC + CMJ at 90° knee flexion + vertical jump max force	30 recreationally active males *Randomised, double-blind, independent groups*	PPT	↓	MIVC	↔
				CK	↔	CMJ	↔
				hsCRP	↔		
Clifford et al. (38)	Beetroot juice^b^ 2 groups: 250 mL OR 125 mL Immediately, 2 h, 24 h and 48 h post-ex	100-drop jumps	30 recreationally active males *Randomised, double-blind, independent groups*	CK	↔	CMJ	↑
						MIVC	↔
Clifford et al. (35)	Beetroot juice^a^ 2 groups: 250 mL OR 125 mL 3 × the day of ex 2 × 24 h post-ex 2 × 48 h post ex	2 × RST 20 × 30 m	20 male team-sports player *Double blind, independent groups*	CK	↔	CMJ	↑
				hs-CRP	↔	RI	↑
				PPT	↑	Sprint time	↔
Martins et al. (40)	Grape juice^a^ 400 mL/d 14 d pre-ex	Volleyball match simulation	12 male volleyball athletes *Randomised, double-blind, crossover, clinical trial*	Lipid peroxidation	↓		
				DNA damage	↓		
Toscano et al. (41)	Grape juice^a^ 10 mL/kg/d 1 d pre-ex	Run to exhaustion at 80% VO_2max_	14 recreational male runners *Randomised, double-blind, crossover, clinical trial*	TAC	↑	TTE	↑
Toscano et al. (39)	Grape juice^a^ 10 mL/kg/d 28 d pre-ex	TTE running test, anaerobic threshold test and aerobic capacity test	28 male and female recreational runners *Randomised, independent groups*	TAC	↑	TTE	↑
				Vitamin A	↑		
				UA	↑		
Ammar et al. (44)	Pomegranate juice^a^ 3 × 250 mL/d 2 d pre-ex 500 mL 1h pre-ex	2 × Olympic-Weightlifting sessions	9 elite weightlifters *Double-blind,crossover*	MDA	↓		
				GPX	↓		
				UA	↓		
Ammar et al. (45)	Pomegranate juice^a^ 3 × 250 mL/d 2 d pre-ex 500 mL 1h pre-ex	2 × Olympic-Weightlifting-sessions	9 elite weightlifters *Double-blind, crossover*	RPE	↓	Performance	↑
				CK	↓		
				HR	↓		
				LDH	↓		
Machin et al. (47)	Pomegranate juice^a^ 2 diff doses:1 × 30 mL/d OR 2 × 30 mL/d (12 h interval) 3 d pre-ex 1 d the day of ex 4 d post-ex	20 min downhill running and 40 max ECC elbow flexion rep.	45 recreationally active males *Randomised, double-blind*	Strength recovery	↑ (both)		
Trombold et al. (16)	Pomegranate juice^a^ 2 × 250 mL (12h interval) 7 d pre-ex 1 d the day of ex (+250 mL immediately after ex) 7 d post-ex	3 × 20 unilateral ECC elbow flexion + 6 ×10 unilateral ECC knee extension at 110% 1RM	17 trained male athletes *Randomised, double-blind, counterbalanced, crossover*	MS	↓ elbow flexors ↔ knee extensor	Isometric	↑ elbow flexor ↔ knee extensor
Trombold et al. (46)	Pomegranate extract^a^ 2 × 500 mL (12 h interval) 4 d pre-ex 1 d the day of ex (+ 500 mL immediately after ex) 4 d post-ex	2 × 20 max ECC elbow flexion (one arm)	16 recreationally active males *Randomised, double-blind, crossover*	MS	↓ (2 h)	Strength	↑ (48 h and 72 h)
				CK	↔		
				Mb	↔		
				Il-6	↔		
				CRP	↔		
McCormick et al. (54)	Tart cherry juice^a^ 90 mL/d 6 d pre-ex	Water-based performance (VJ, 10 m ST, RST and WIST)	9 male Water Polo athletes *Randomised, double-blind, crossover, repeated measure*	IL-6	↔	Performance	↔
				CRP	↔		
Howatson et al. (15)	Tart cherry juice^a^ 2 × 237 mL/d 5 d pre 1 d the day of ex 2 d post-ex	Marathon run	20 (13 males and 7 females) recreational marathon runners *Double-blind, independent groups*	CK	↔	MVIC	↑
				LDH	↔		
				DOMS	↔		
				TAS	↑		
				TBARS	↓		
				PC	↔		
				CRP	↓		
				IL-6	↓		
				UA	↓		
Kuehl et al. (48)	Tart cherry juice^a^ 2 × 355 mL/d 7 d pre-ex 1 d the day of ex	Hood to Coast Relay (~28h) total distance: 22.5 km–31.4 km	54 (36 males and 18 females) healthy runners *Randomised, double-blind*	VAS	↓		
Bell et al. (51)	Montmorency cherry juice^a^ 2 × 30 mL/d 4 d pre-ex 1 d the day of ex 3 d post-ex	109 min cycling trial	16 male trained cyclists Double-blind, counterbalanced, independent groups	hsCRP	↓	MVIC	↑
				IL-6	↓		
Bell et al. (50)	Montmorency cherry juice^a^ 2 × 30 mL/d 4 d pre-ex 3 d the day of ex	3 d 109 min cycling trial	16 male trained cyclists *Double-blind, counterbalanced, independent groups*	IL-6	↓		
				hsCRP	↓		
Bowtell et al. (52)	Montmorency cherry juice^a^ 2 × 30 mL/d 7 d pre-ex 1 d the day of ex 2 d post-ex	10 × 10 single-leg knee extension at 80% 1RM	10 well-trained male athletes *Randomised, double-blind, crossover*	CK	↔	MVC	↑
				PPT	↔		
				TAS	↔		
				PC	↓		
				hsCRP	↔		
Boussetta et al. (54)	Red orange juice^a^ 500 mL 2.5 h pre-ex	YYIRT-1	11 male healthy soccer players *Randomised, single-blind, crossover*	CK	↓		
				MDA	↓		
Lima et al. (59)	Anthocyanin-rich juice^a^ (apple + prum + blueberry + maquiberry + raspberry + cranberry) 2 × 240 mL/d (12h interval) 4 d pre-ex 1 d the day of ex 4 d post-ex	30 min DHR at 70% VO_2max_ speed	30 healthy young males *Randomised, double-blind, independent group*	MS	↓	RE	↑ (24 h)
				CK	↓		
Lynn et al. (60)	Bilberry juice^a^ 2 × 200 mL/d 5 d pre-ex 1 d the day of ex 2 d post-ex	Half-marathon	21 (16 males and 5 females) recreationally trained runners *Randomised single-blind, independent group*	CRP	↑		
				CK	↑		
				DOMS	↑		
Sadowska-kr pa et al. (55)	Acai berry-based juice^c^ 100 mL/d 42 d (ex was done before and after supplementation period)	300 m sprint running	7 junior hurdlers *Pre- and post-comparison*	CK	↓	Performance	↔
				LDH	↓		
Tsitsimpikou et al. (58)	Tomato juice^a^ 100 g 2 months during and post-ex	2 months anaerobic training	15 anaerobically trained athletes *Randomised, independent group*	CPK	↓		
				LDH	↓		
				CRP	↓		
Tarazona-Díaz et al. (57)	Watermelon juice^a^ 500 mL 1 h pre-ex	11 min resistance (4.5 kg–5 kg) exercise using cycle ergometer	7 male Sports Science students *Randomised, crossover*	RPE	↔		
				MS	↓		
Mcleay et al. (56)	New Zealand blueberry^a^ 200 g blueberry + 50 g banana + 200 mL apple juice 5 h and 10 h pre-ex, immediately, 12 h and 36 h after ex	3 × 100 single-knee ECC flexion 30°.s^−1^	10 recreationally active females *Randomised, crossover*	CK	↔	Isometric	↑
				MS	↔	ECC	↔
				PC	↔	CON	↔
				ROS-GC	↓		
				FRAP	↑		
				IL-6	↔		

**Abbreviations:** Pre-ex = pre-exercise; Post-ex = post-exercise; RCT = randomised controlled trial; CK = creatine kinase; DOMS = delayed-onset muscle soreness; Mb = serum myoglobin; LDH = lactate dehydrogenase; GPX = glutathione peroxidase; UA = uric acid; HR = heart rate; MS = muscle soreness; CPK = creatine phosphokinase; TAS = total antioxidant status; TAC = total antioxidant capacity; PC = protein carbonyls; IL = interleukin; hsCRP = high-sensitivity C-reactive protein; MDA = malondialdehyde; PPT = pressure-pain threshold; VAS = visual analog scale; TC = thigh circumference; MF = impaired muscle function; TTE = Time-to-exhaustion; PIMT = peak isometric torque; PIKT = peak isokinetic torque; RPE = rating of perceived exertion; MVC = maximal voluntary contractions; MIVC = maximal isometric voluntary contractions; CMJ **=** countermovement jumps; SJ = squat jump; RI = reactive strength index; RM = repetition maximum; DHR = downhill running; RE = running economy; LIST = Loughborough Intermittent Shuttle Test; VJ = in-water vertical jump test; ST = sprint test; RST = repeated sprint test; WIST = Water Polo intermittent shuttle test; YYIRT-1 = Yo-Yo Intermttent Recovery Test Level-1; VO_2ma_ = maximal oxygen consumption

↓ significant decrease/lower; ↑ significant increase/higher; ↔ no significant change/no significant difference

a Comparison with a placebo/control group

b Comparison with sodium nitrate and placebo group

c Comparison between before and after consumption of supplement
